# Patients’ self-triage for unscheduled urgent care: a preliminary study on the accuracy and factors affecting the performance of a Belgian self-triage platform

**DOI:** 10.1186/s12913-022-08571-5

**Published:** 2022-09-23

**Authors:** Allison Gilbert, Anh Nguyet Diep, Maryame Boufraioua, Benoit Pétré, Anne-Françoise Donneau, Alexandre Ghuysen

**Affiliations:** 1 Emergency Department, University Hospital Center, Avenue de L’Hôpital 1, 4000 Liège, Belgium; 2 Public Health Department, University of Liège, Quartier Hôpital, Av. Hippocrate 13, CHU B23, 4000 Liège, Belgium; 3 Biostatistics Unit, University of Liège, Quartier Hôpital, Av. Hippocrate 13, CHU B23, 4000 Liège, Belgium

**Keywords:** Self-triage, Unscheduled care, Emergency department, Interactive platform

## Abstract

**Background:**

Management of unscheduled urgent care is a complex concern for many healthcare providers. Facing the challenge of appropriately dispatching unscheduled care, primary and emergency physicians have collaboratively implemented innovative strategies such as telephone triage. Currently, new original solutions tend to emerge with the development of new technologies. We created an interactive patient self-triage platform, ODISSEE, and aimed to explore its accuracy and potential factors affecting its performance using clinical case scenarios.

**Methods:**

The ODISSEE platform was developed based on previously validated triage protocols for out-of-hours primary care. ODISSEE is composed of 18 icons leading to algorithmic questions that finally provide an advised orientation (emergency or primary care services). To investigate ODISSEE performance, we used 100 clinical case scenarios, each associated with a preestablished orientation determined by a group of experts. Fifteen volunteers were asked to self-triage with 50 randomly selected scenarios using ODISSEE on a digital tablet. Their triage results were compared with the experts’ references.

**Results:**

The 15 participants performed a total of 750 self-triages, which matched the experts references regarding the level of care in 85.6% of the cases. The orientation was incorrect in 14.4%, with an undertriage rate of 1.9% and an overtriage rate of 12.5%. The tool’s specificity and sensitivity to advise participants on the appropriate level of care were 69% (95% CI: 64—74) and 97% (95% CI: 95—98) respectively. When combined with advice on the level of urgency, the tool only found the correct orientation in 68.4% with 9.2% of undertriages and 22.4% of overtriages. Some participant characteristics and the types of medical conditions demonstrated a significant association with the tool performance.

**Conclusion:**

Self-triage apps, such as the ODISSEE platform, could represent an innovative method to allow patients to self-triage to the most appropriate level of care. This study based on clinical vignettes highlights some positive arguments regarding ODISSEE safety, but further research is needed to assess the generalizability of such tools to the population without equity issues.

**Supplementary Information:**

The online version contains supplementary material available at 10.1186/s12913-022-08571-5.

## Introduction

For years, unscheduled urgent primary and emergency care management has been considered a complex task for healthcare practitioners. Emergency and primary care physicians are both facing complex management issues resulting from overwhelming workloads. Indeed, the demand for unscheduled urgent care is still growing, whereas available physicians are lacking, and more appropriate organizational structures need to be implemented to improve emergency care regulation [1]. Such dilemmas have led to deleterious consequences such as more stressful working conditions, impaired quality of care, physicians’ burnout, and overall reduced patient safety [2, 3]. The accurate triage of patients in need of unscheduled care and their orientation to the most appropriate location at the right time still represent challenging work for healthcare professionals [1]. Several strategies intended to organize this rather chaotic situation have been implemented to guide patients to the most appropriate level of care before any medical contact. Among these, nurse telephone triages and other dispatching helplines have emerged as valid solutions but require specifically trained and experienced clinical or nonclinical dispatchers [4, 5].

The establishment of digital and Internet-based services led to opportunities in the organization of the healthcare system. The availability of medical information on the Internet and the widespread use of mobile computers, digital tablets and smartphones have led patients to search for healthcare information on the Internet before considering any medical contact. However, many studies have demonstrated that most patients seeking for medical advice on the Internet do not fully consider how secure or valid the advising platforms could be [6, 7]. Although a more recent study by *Levine D et al.* found that such a searching initiative may not be as harmful as previously thought in terms of diagnosis accuracy, they did not identify an association with triage accuracy [8]. Facing the rise of the digital area, medical experts intended to propose an innovative dispatching strategy through informative and guidance tools for the patients to self-triage.

The increasing interest in developing and implementing accurate interactive tools to safely guide patients in need of unscheduled urgent care is directly linked to the concept of patient empowerment. As *Alfano M et al.* reported in their recent article, patient empowerment is based on three concepts: promoting understanding, improving the informed decision process, and enhancing self-management [9]. Technological applications can empower patients in two ways: better decision-making and better acting [9]. However, some authors are more skeptical regarding the impact of such empowerment self-triage tools on health services utilization and their concrete safety [10–12].

Two key aspects are relevant when assessing interactive healthcare platforms, i.e., the intention to use and the platform’s usability. Recent research has highlighted the factors that might affect the intention to use [13] but few have focused on their appropriate usability considering specific characteristics, notably age, gender, the habit of using digital devices, education level or health literacy [9]. Usability is a complex concept to investigate as it encompasses multiple factors. The International Standard ISO 9241–11 defines *usability* as “the extent to which a product can be used by specified users to achieve specified goals with effectiveness, efficiency and satisfaction in a specified context of use” [14]. Effectiveness is one component of usability and is directly linked to the concept of accuracy to achieve the required goals [15].

We developed a self-triage platform to guide patients in need of unscheduled urgent care to the best level of care. The mobile app ODISSEE (*Outil Décisionnel et Informatif des Structures de Soins Efficientes Existantes*) was developed based on reliable and valid triage protocols for out-of-hours primary care [5, 16–18]. In the present preliminary study, we aim to investigate the usability of this self-triage prototype assessing the ODISSEE accuracy using simulated clinical case scenarios and exploring whether potential patient characteristics could favorably or negatively affect the tool’s performance.

## Methods

### The ODISSEE platform

The ODISSEE platform (*Outil Décisionnel et Informatif des Structures de Soins Efficientes Existantes*) is a French-language interactive app that allows patients to self-triage and obtain knowledgeable advice regarding the most appropriate level of care for their current condition. The app’s prototype was based on reliable and valid triage protocols for out-of-hours primary care, the SALOMON algorithms. Those protocols have been experienced in our hospital center for five years with favorable results regarding patient safety and satisfaction [5, 16–18].

**Fig. 1:**
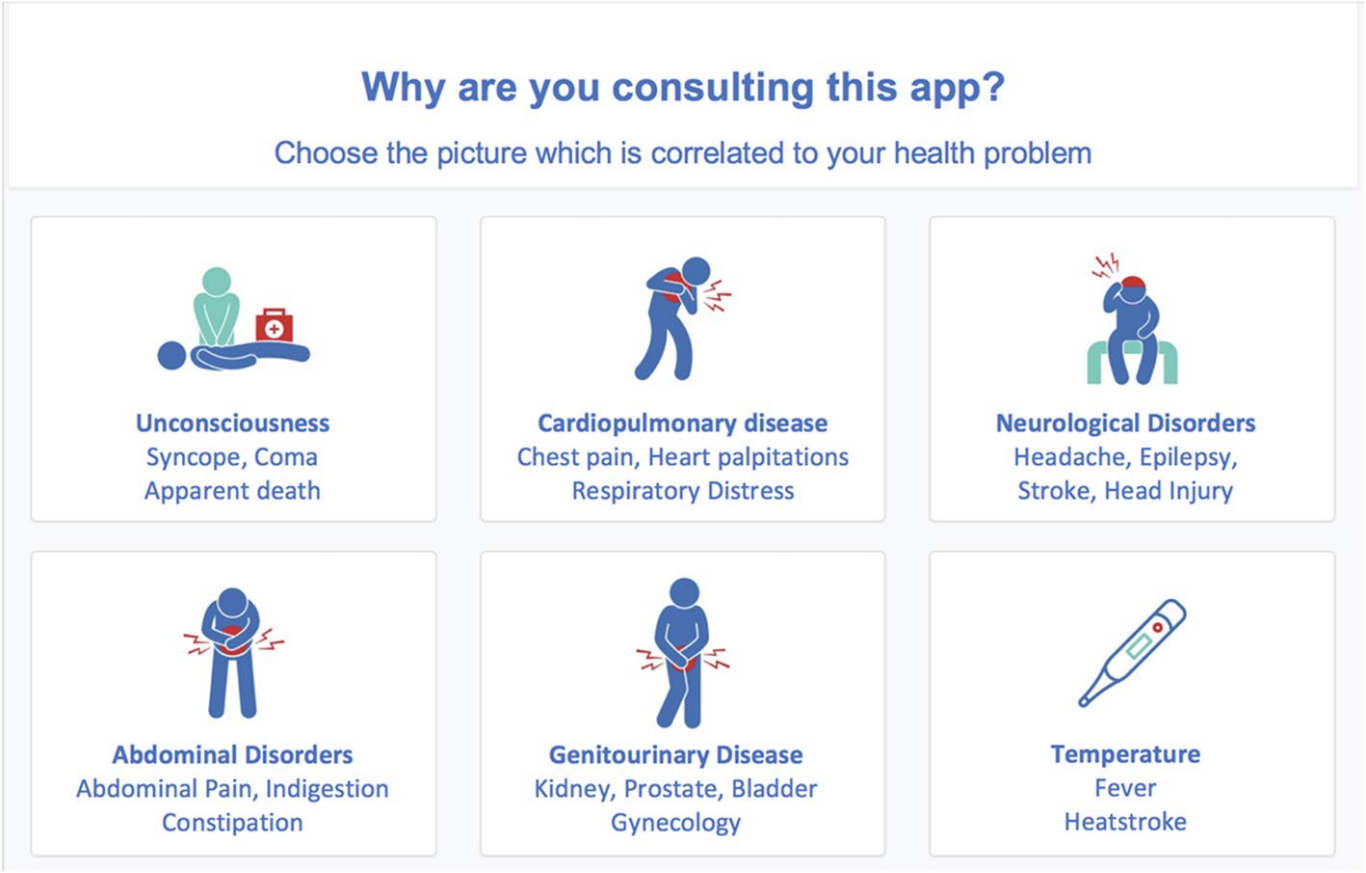
Presentation of the different pictures of the ODISSEE platform related to the most frequent pathologies encountered in the unscheduled care settings

The prototype comprises 18 icons corresponding to the most frequently encountered conditions in the unscheduled care settings (Fig. 1). The patients are invited to choose the most appropriate icon regarding their complaints and are then directed to different algorithmic flowcharts leading them to a final advice of referral.

The advice provided is sorted out of four potential referrals or triage categories based on the levels of care required and the levels of urgency. These four referrals have been distinguished into two levels of care: emergency departments versus primary care services. Furthermore, each level of care is associated with two degrees of urgency for each category: Emergency Level 1 (Emergency Medical Services) versus Emergency Level 2 (Emergency Department Referred Consultation) and Primary Care Level 1 (Primary Care Physician Immediate Visit) versus Primary Care Level 2 (Primary Care Physician Delayed Visit). The referrals are detailed in Table 1.

**Table 1:** Four referrals proposed by the ODISSEE platform with the 2 different levels of care and their associated levels of urgency

**Level of urgency**	**Level of care**	
	***Emergency Departments***	***Primary Care Services***
***Level 1***	**Emergency Medical Services** **112 European Emergency Number** *(EMS-Emergency Level 1)* The patient is advised to immediately contact the 112-dispatching center	**Primary Care Physician** **Immediate Consultation** *(PCPI-Primary Care Level 1)* The patient is advised to call the primary care physician on duty
***Level 2***	**Emergency Department** **Referred Consultation** *(EDRC-Emergency Level 2)* The patient is advised to attend an Emergency Department	**Primary Care Physician** **Delayed Consultation** *(PCPD-Primary Care Level 2)* The patient is advised to schedule a consultation in a primary care facility

### Study design

We conducted a prospective preliminary study to assess the accuracy of the ODISSEE platform. We used 100 clinical case scenarios created by a group of emergency physicians, general practitioners and emergency nurses [16, 17]. These clinical cases were adapted to fit with the use of an interactive mobile app (see example in Supplementary Files – Supplementary File S1). The 100 scenarios covered all the algorithms used to create the ODISSEE tool. Those scenarios mainly encompassed the most frequent symptoms encountered in the unscheduled care settings. Each scenario was associated with a particular preestablished theoretical triage level assigned by a group of experts (2 emergency physicians, members of the local general practice cooperatives and 3 emergency nurses). In the clinical vignettes, all the information required for self-triage was given including age, sex, history of the disease and symptoms. If the vignette required the knowledge of a specific clinical particularity, a picture was provided (e.g., rashes in case of dermatological diseases or wound in case of specific trauma vignettes, etc.).

### Characteristics of the scenarios

For the study, 100 scenarios were used. Among those, 62% (*n* = 62) were categorized in the Emergency Level, of which 59.7% (*n* = 37) were classified in Emergency Level 1 and 40.3% (*n* = 25) in Emergency Level 2. Likewise, 38% (*n* = 38) of the scenarios were assigned as Primary Care Level, of which 68.4% (*n* = 26) were at Primary Care Level 1 and 31.6% (*n* = 12) at Primary Care Level 2. There are different types of scenarios designed which were grouped as found in Table 2.

**Table 2:** The type of scenarios per design and the respective number (percentage) (*N* = 100)

Type of scenarios (*N* = 100)	Number
Dermatological diseases	11
Trauma	25
Digestive/Abdominal diseases and associated surgery complications	9
Cardiac and pulmonary diseases	9
Intoxication and psychiatric problems	7
Neurological disorders	10
Others^a^	29

^a^ Minor diabetes complications, pregnancy regular problems, ocular and ear-nose-throat disorders, frequent pediatric disorders, problem involving > 5 patients, fever, non-traumatic articular conditions

### Study settings and population

The study was conducted by two investigators from the University Hospital Center of Liège. An advertisement for the study was performed by the investigators in charge of selecting appropriate volunteers for the test. Inclusion criteria to be included in the study were as follows: all male and female adults, with no medical or paramedical expertise, with the ability to use a digital tablet and to fully understand French language. Children (age < 18 years old) and adults with medical or paramedical training were refuted to participate in the study. After the recruitment phase, only 15 participants who matched the inclusion criteria were included in the study. Different participant characteristics were collected: age, gender, educational degree, and interest in health information found on the internet.

Among the 100 scenarios, because of the feasibility for each participant to test more than 50 scenarios, two sets of 50 scenarios were randomly created. Each participant randomly received one set to test. An appointment was scheduled between the investigator and the volunteer for participation in the study. Participants were asked to triage themselves using the ODISSEE app on a digital tablet. The participants were not informed of the pre-established theoretical triage given by the experts. The investigators did not take part in the self-triage and were not allowed to interfere with the assessment. The participants were not submitted to time pressure. After the self-triage, the investigators compared the application referral with the theoretical triage.

### Definitions of accuracy, levels of care and urgency

In this study, three objectives were addressed, i.e., evaluating the accuracy of the tool, assessing the potential factors affecting the accuracy, and focusing on the error rate, overtriages and undertriages. For these purposes, we defined different concepts required for the analysis. Accuracy was defined as the ability of the tool to provide an appropriate advice regarding the triage category (level of care plus level of urgency, yielding a total of four referrals or triage categories), the level of care alone or the level of urgency alone. Regarding inappropriate advice, overtriage was defined as an advice recommending a higher level of care or urgency and undertriage was defined as a destination with a lower level of care or urgency compared to the gold standard defined for the scenario.

As previously described in Table 1, to further consider the analysis regarding the appropriate level of care, the emergency care level was defined by combining the EMS-Level 1 and EDRC-Level 2. Likewise, the primary care level was considered by combining the PCPI-Level 1 and PCPD-Level 2.

In analyzing the appropriate level of urgency, we considered the comparison between Emergency Level 1 versus Emergency Level 2 and Primary Care Level 1 versus Primary Care Level 2.

We examined the performance of the tool to advise the participant on the correct level of care and urgency (triage category) on a total number of 750 triages provided by the participants. Subsequently, we investigated the capacity to address the participant to the appropriate level of care (Emergency Departments versus Primary Care Services) with a sample size of 750 triages and to advise the participant on the adequate level of urgency (Level 1 versus Level 2) with sample sizes of 432 (Emergency Care Level 1 vs Level 2) and 210 (Primary Care Level 1 vs Level 2) due to the false classifications by the participants.

### Ethics declarations

The study followed the declaration of Helsinki principles and was approved by the ethics committee of the University Hospital Center of Liège (ref. 2021–226). Informed consent was obtained from all participants to the study before the investigation.

### Statistical analysis

The results have been encoded in a database and anonymized directly from the digital tablet. First, descriptive statistics were performed such that the results were expressed as medians and interquartile ranges (IQRs) for quantitative variables and as counts and proportions (%) for qualitative variables. Second, the performance of the tool vs. the theoretical decisions (gold standard) across the 750 cases was evaluated using percentage of agreement and Fleiss’ kappa for non-fully crossed design, in addition to indicators including sensitivity, specificity, positive and negative predictive values with the corresponding 95% confidence intervals (95% CI). The analysis was conducted for the pooled sample (*N* = 750) and for each level within the Emergency and Primary Care categories. Third, for each type of scenarios, the percentage of agreement between the tool and the gold standard and Fleiss’ kappa were reported with corresponding 95% CI. Fleiss chi-squared was employed to test the equality of kappa values across different types of scenarios.

Finally, the percentage of agreement and the extent of agreement between the participants’ self-triage and the gold standard were examined as a function of age, educational level, and interest in health information search on the internet. Hotelling’s T-squared (T^2^) was used to test the difference in agreement among different groups of participants. The analyses were performed using R (R Core Team, 2013).

## Results

### Characteristics of the participants

The median age of the participants was 37 years old [IQR: 26.00 – 64.50] (range: 25–75 years old) with a sex-ratio of 0.9 (7/8). Among the 15 participants, 26.7% (*n* = 4) followed a secondary education, 20% (*n* = 3) followed a non-university higher education and finally, 53.3% (*n* = 8) of the participants followed a university formation. For the purpose of analysis, the secondary and non-university degree holders were collapsed into one group (*n* = 7, 46.67%).

Regarding the interest in health information found on the internet, 53.3% (*n* = 8) of the people were interested whereas 46.7% (*n* = 7) were not. These characteristics are depicted in Table 3.

**Table 3:** Characteristics of the 15 participants

Characteristics	Participants of the study (*N* = 15)
***Age (year)***	
Median (IQR)	37.00 (IQR: 26.00 – 64.50)
***Gender***	
Male	7 (46.7%)
Female	8 (53.3%)
***Level of education***	
Primary education	0 (0%)
Secondary education	4 (26.7%)
Higher education, non-university	3 (20%)
Higher education, university	8 (53.3%)
***Interest in health information on the internet***	
Yes	8 (53.3%)
No	7 (46.7%)

### Self-triage accuracy by participants compared to the gold standard (Table 4)

**Table 4:** The sensitivity, specificity, and positive and negative predictive values of the classification by the tool and the gold standard

	**Level of care (** ***N*** ** = 750)**	**Level of urgency (** ***n*** ** = 642)**	
**Criteria**	**Emergency vs. Primary care**	**Emergency care (1 vs. 2)** **(** ***n*** ** = 432)**^**a**^	**Primary care (1 vs. 2)** **(** ***n*** ** = 210)**^**a**^
Sensitivity	0.97 (0.95—0.98)	0.83 (0.78—0.87)	0.91 (0.85—0.95)
Specificity	0.69 (0.64—0.74)	0.72 (0.65—0.79)	0.65 (0.53—0.76)
Positive predictive value	0.82 (0.79—0.85)	0.81 (0.76—0.86)	0.83 (0.77—0.89)
Negative predictive value	0.94 (0.90—0.97)	0.74 (0.67—0.81)	0.80 (0.67—0.89)
% Agreement	85.6%	78.7%	82.4%
Fleiss’ kappa	0.687 (0.604—0.770)	0.552 (0.446 – 0.659)	0.583 (0.399 – 0.768)

^a^ Sample size was adjusted after removing falsely classified cases by the participants

#### Triage categories

Among the 750 self-triages encoded by the 15 participants, the accuracy of the tool to adequately define both appropriate level of care and level of urgency was found in 68.4% (95% CI: 0.63 – 0.74) of the cases (*n* = 513) with a Fleiss’ kappa = 0.557 (0.510 – 0.605).

#### Emergency and primary care

The percentage agreement between participants’ self-triage and the gold standard in deciding the need for emergency care versus primary care was 85.6%. Fleiss’ kappa = 0.687 (0.604—0.770) revealed substantial agreement next to a very high sensitivity of 97% (0.95—0.98). The specificity value was 69% (0.64—0.74), and the positive predictive value (PPV) and negative predictive value (NPV) were 82% (0.79—0.85) and 94% (0.90—0.97), respectively.

#### Levels of emergency care and primary care

The participants’ classification of Emergency Care level 1 against level 2 showed a 78.7% (0.744 – 0.831) agreement with the gold standard (*n* = 432, removing falsely classified cases). Fleiss’ kappa = 0.552 (0.446 – 0.659) indicated moderate agreement. The sensitivity and specificity to predict the need for emergency care were 0.83 (0.78—0.87) and 0.72 (0.65—0.79), respectively. The positive predictive value (PPV) and negative predictive value (NPV) were estimated at 0.81 (0.76—0.86) and 0.74 (0.67—0.81), respectively.

Regarding the referral to Primary Care levels, the participants’ classification revealed an 82.4% agreement with the gold standard with Fleiss’ kappa = 0.583 (0.399 – 0.768) indicating moderate agreement. Accordingly, the sensitivity and specificity of the participants’ self-triage to appropriately refer to Primary Care Services were evaluated to be 0.91 (0.85—0.95) and 0.65 (0.53—0.76) respectively, with a PPV of 0.83 (0.77—0.89) and an NPV of 0.80 (0.67—0.89).

### Assessment of potential factors affecting the accuracy

#### Impact of the participants’ characteristics on the accuracy

To compare the self-triage performance of the participants across different groups, a quota selection such that each of the scenarios was triaged by a fixed number of participants representing each group was carried out.

##### Age

Accordingly, one scenario was triaged by 6 participants from three age groups and 6 participants from 2 educational level groups (non-university vs. university degree holders). Fifty scenarios were triaged by 6 participants and another 50 scenarios were triaged by 8 participants from two groups showing interest or non-interest in health information searches on the internet.

In the group of participants ranging from 18- to 25- year-old (*n* = 4), 39% (*n* = 78) of triage errors were made. Among those of 26- to 50- year-old (*n* = 4), 21.5% (*n* = 43) of triage errors were noted. Finally, in the 51- to 75- year-old group (*n* = 7), 33.4% of errors were noticed.

The results revealed that the percentage of agreement with the gold standard was highest in the group between 26- and 50-year-old with 78.5% being found, followed by the group of 51 to 75 years (68.5%). The least agreement was found in the self-triage of the 18- to 25-year-old group (61.0%). Pairwise comparisons revealed that Fleiss’ kappa was significantly higher in the 26- and 50-year-old age groups with a value of 0.70 (0.62–0.77) indicating substantial agreement compared to that from the other two groups. No significant difference in the extent of agreement with the gold standard was found between the self-triage of the 18- to 25-year-old and the 51- to 75-year-old groups (T^2^ = 3.71, *p* = 0.057).

##### Education degree and interest in digital health information search

Regarding the education level, the participants with a university education (*n* = 8) made 31.2% (125) of errors while those with no university education (*n* = 7) made 32.3% (*n* = 113) errors in self-triage scenarios. Participants who were interested in health information found on the internet (*n* = 8) performed no better than those who were not (*n* = 7) with 31.8% (*n* = 127) and 31.7% (*n* = 111) of misdirections advised by the self-triage, respectively.

A slightly higher percentage of agreement (71%) was found in the self-triage of the university degree holders’ group with Fleiss’ kappa = 0.59 (0.51 – 0.67) indicating moderate agreement compared to that of the non-university degree holders (66.30%), Fleiss’ kappa = 0.52 (0.44 – 0.61). However, Hotelling’s T-squared test revealed a nonsignificant difference in the extent of agreement, T^2^ = 1.56, *p* = 0.215. A similar result was found for participants who were interested in health information found on the internet and those who were not, T^2^ = 0.43, *p* = 0.516 with both groups displaying moderate agreement with the gold standard. The results are presented in Table 5.

**Table 5:** Percentage of agreement and Fleiss’ kappa for the self-triage by the participants compared with the gold standard across different age, education levels, and interest in health information search

**Age groups** **(number of self-triages = 600)**	**Percentage of agreement**	**Fleiss’ kappa**	**Pairwise** **comparison**	**Hotelling’s T-squared**
Group 1: 18 to 25 years	61.00%	0.44 (0.34 – 0.55)	18 to 25 years vs. 26 to 50 years	T^2^ = 17.00, *p* < 0.001
Group 2: 26 to 50 years	78.50%	0.70 (0.62 – 0.77)	26 to 50 years vs. 51 to 75 years	T^2^ = 6.82, *p* = 0.010
Group 3: 51 to 75 years	68.50%	0.55 (0.47 – 0.64)	18 to 25 years vs. 51 to 75 years	T^2^ = 3.71, *p* = 0.057
**Educational level** **(number of self-triages = 600)**	**Percentage of agreement**	**Fleiss’ kappa**	**Pairwise** **comparison**	**Hotelling’s T-squared**
Non-university	66.30%	0.52 (0.44 – 0.61)	Non-university vs. university degree holders	T^2^ = 1.56, *p* = 0.215
University degree holders	71%	0.59 (0.51 – 0.67)		
**Interest in health information search on Internet** **(number of self-triages = 700)**	**Percentage of agreement**	**Fleiss’ kappa**	**Pairwise** **comparison**	**Hotelling’s T-squared**
Non-interest	68.6%	0.57 (0.49 – 0.65)	Non-interest vs. interest in health information search on Internet	T^2^ = 0.43, *p* = 0.516
Interest	66.90%	0.54 (0.46 – 0.62)		

#### Influence of the type of scenarios

Based on the design, we grouped the 100 scenarios into 7 types, including “dermatological diseases”, “trauma”, “digestive/abdominal diseases and associated surgery complications”, “cardiac and pulmonary diseases”, “intoxication and psychiatric disorders”, “neurological disorders”, and “others” (minor diabetes complications, pregnancy regular problems, ocular and ear-nose-throat disorders, frequent pediatric disorders, non-traumatic articular conditions, problems involving more than 5 patients, isolated fever).

As shown in Table 6, in general, the participants’ self-triage of 4 levels of care combined (2 levels within Emergency Care and 2 levels within Primary care) showed a moderate agreement with the gold standard, Fleiss’ kappa = 0.56 (0.49- 0.62) and a 68.4% of agreement.

**Table 6:** The percentage of agreement, Fleiss’ kappa, and pairwise comparisons across different types of scenarios

Type of scenarios	n	% agreement	Kappa (95% CI)	Pairwise comparisons^a^		*p*-value
Dermatological diseases	84	61.90%	0.46 (0.30—0.61)	Digestive/Abdominal diseases and associated surgery complications vs	Dermatological diseases	0.005
Trauma	185	68.60%	0.51 (0.38—0.634)		Trauma	0.015
Digestive/abdominal diseases and associated surgery complications	68	83.80%	0.75 (0.62—0.89)		Cardiac and pulmonary diseases	0.013
Cardiac and pulmonary diseases	68	63.20%	0.41 (0.18—0.65)		Intoxication and psychiatric problems	0.014
Intoxication and psychiatric problems	51	74.50%	0.47 (0.29—0.65)		Neurological disorders	0.000
Neurological disorders	73	53.40%	0.30 (0.12—0.48)	Neurological disorders vs	Others	0.006
Others^b^	221	71.00%	0.60 (0.48—0.72)			
Overall	750	68.40%	0.56 (0.49- 0.62)			0.002

^a^ Only significant differences are presented

^b^ Including the “Others” category

The results showed that the most agreement occurred with scenarios related to “digestive/abdominal diseases and associated surgery complications” (83.8%), “intoxication and psychiatric disorders” (74.5%), and the “others” (71.0%) and the least agreement was found in scenarios associated with “neurological disorders” (53.40%) and “dermatological diseases” (61.9%). After correction for chance, the results indicated that scenarios of “digestive/abdominal diseases and associated surgery complications” and the “others” observed the highest Fleiss’ kappa. The omnibus Fleiss chi-squared revealed a significant difference in the agreement indexes, χ^2^ = 20.616, *p* = 0.002. Post-hoc analyses suggested that the agreement with the gold standard was significantly higher in the scenario type “digestive/abdominal diseases and associated surgery complications” compared with those of “dermatological diseases”, “trauma”, “cardiac and pulmonary diseases”, “intoxication and psychiatric disorders”, and “neurological disorders”. Additionally, the extent of agreement between participants’ self-triage was significantly lower in “neurological disorders” scenarios compared to those labelled “others”. The results are illustrated in Table 6 with only significant post-hoc pairwise comparisons being displayed.

### Error rate, overtriage and undertriage (Table 7)

**Table 7:** Different errors regarding both levels of care and urgency (4 categories of triage), level of care alone (Emergency vs Primary care) and level of urgency alone (Level 1 vs Level 2)

**Triage Category**	**Triage Category**			**Level of care**			**Level of urgency**		
	***N*** ** = 750**			***N*** ** = 750**			***N*** ** = 642**^**a**^		
	Appropriate	Undertriage	Overtriage	Appropriate	Undertriage	Overtriage	Appropriate	Undertriage	Overtriage
**EMS**	*N* = 213 (28.4%)		*N* = 82 (10.9%)	*N* = 432 (57.6%)		*N* = 94 (12.5%)	*N* = 213 (33.2%)		*N* = 49 (7.6%)
**EDRC**	*N* = 127 (16.9%)	*N* = 43 (5.8%)	*N* = 61 (8.1%)				*N* = 127 (19.8%)	*N* = 43 (6.7%)	
**PCPI**	*N* = 126 (16.8%)	*N* = 13 (1.7%)	*N* = 25 (3.4%)	*N* = 210 (28%)	*N* = 14 (1.9%)		*N* = 126 (19.6%)		*N* = 25 (3.9%)
**PCPD**	*N* = 47 (6.3%)	*N* = 13 (1.7%)					*N* = 47 (7.3%)	*N* = 12 (1.9%)	
**TOTAL**	*N* = 513 (68.4%)	*N* = 69 (9.2%)	*N* = 168 (22.4%)	*N* = 642 (85.6%)	*N* = 14 (1.9%)	*N* = 94 (12.5%)	*N* = 513 (79.9%)	*N* = 55 (8.6%)	*N* = 74 (11.5%)

^**a**^ Sample size was adjusted after removing falsely classified cases by the participants

#### Determining the level of care

Regarding the level of care needed (*n* = 750), the application had an error rate of 14.4% (95% CI: 0.08 – 0.21) (*n* = 108) with an overtriage rate of 12.5% (*n* = 94) and an undertriage rate of 1.9% (*n* = 14).

Among the 14 participants who were undertriaged as requiring primary care instead of emergency care (1.9%), 13 participants (1.7%) were categorized as PCPI and 1 (0.13%) was categorized as PCPD.

Among the 94 participants overtriaged to emergency services instead of primary care services (12.5%), 33 participants (4.4%) were classified as EMS and 61 (8.1%) as EDRC.

#### Determining the level of urgency

Regarding the level of urgency (*n* = 642), the application gave mistaken advice in 20.1% (95% CI: 0.13 – 0.27) of the cases (*n* = 129).

Among those errors, 74 participants (11.5%) were overtriaged and 55 (8.6%) were undertriaged.

Regarding the undertriages (*n* = 55), 12 participants were categorized as PCPD (1.9%) instead of PCPI and 43 participants were classified as EDRC (6.7%) instead of EMS.

Concerning the overtriages (*n* = 74), 25 participants were advised as PCPI (3.9%) instead of PCPD and 49 participants (7.6%) were advised as EMS instead of EDRC.

#### Determining the triage category

The precision of the application to provide the appropriate level of urgency associated with the level of care was lower with an error rate of 31.6% (95% CI: 0.25 – 0.37) (*n* = 237) composed of an overtriage rate of 22.4% (*n* = 168) and an undertriage rate of 9.2% (*n* = 69).

#### Error rates associated with the types of scenarios

The distribution of the errors among the different groups of scenarios regarding the triage category, the level of care and the level of urgency is detailed in Supplementary Files – Supplementary File S2.

## Discussion

### Questioning the accuracy of self-triage platforms

Implementing self-triage platforms represents a complex challenge in terms of accuracy, safety and equity. Indeed, technological innovations still generate many debates on their potential positive and negative aspects in the healthcare system. Digital triage tools are mainly criticized for their uncertain accuracy in appropriately delivering medical information, even if most researchers agree on the evident benefit of advertised healthcare information [19, 20]. Moreover, the proper understanding of the given information through web-based applications also remains hardly assessable, leading to many fears from healthcare providers. Currently, technological advances have been integrated into healthcare daily practice and patients are no strangers to this development. Indeed, patients seem prone to use Internet-based services and applications to be empowered in their health management, leading progressively to an actual healthcare provider-patient partnership. This goal is often made difficult by overly sophisticated platforms that use medical jargon or the application’s unattractive layout, whereas patients appreciate easier, understandable and more patient-centered platforms [21]. Additionally, different types of self-assessment platforms exist, those that provide diagnosis, advice, or a potential orientation compared to those mainly focusing on determining the level of care needed. The interest for one or another remains unclear. All these different criteria make the benefits of such e-health interventions challenging to predict and further research is necessary to clarify the potential improvement expected for the healthcare system [22, 23]. In this preliminary work, our results regarding the ODISSEE prototype accuracy tend to demonstrate promising findings in terms of patients’ orientation to the best level of care and suggest perspectives for further research and developments of digital self-triage tools.

### Accuracy of the ODISSEE application to offer appropriate self-triage advice

Management of unscheduled primary and emergency care is a complex concern in which e-health innovations might have some potential to provide original improvements. Recently, due to the COVID-19 pandemic but not specifically, self-assessment and triage platforms have emerged in many countries as a new alternative to triage services supported by healthcare leaders [24, 25].

Based on the present study’s results, the ODISSEE platform seems to offer new opportunities to develop an accurate self-triage tool to guide the patient to the best level of care (85.6% of the cases) with a sensitivity and specificity, estimated at 97% and 69%, respectively.

The ODISSEE platform demonstrates a satisfactory accuracy in referring the patient to the level of care needed (emergency services or primary care services) with a low undertriage rate (1.9%). In view of the level of urgency among these levels of care, the accuracy percentages remain adequate (68.4% of the cases) although it seems to represent a more difficult variable to accurately determine. Comparing self-triage apps to each other is particularly difficult due to the heterogeneity of their structure but currently, some of these tend to propose a high level of performance [26]. In accordance with the Dutch self-triage app “Moet ik naar the dokter”, the ODISSEE tool is also based on validated telephone triage protocols [27]. The sensitivity and specificity of the ODISSEE tool demonstrated higher sensitivity but a slightly lower performant specificity than their application, which estimated sensitivity and specificity values of 84% and 74%, respectively [28]. However, the Dutch results were based on a real-life settings study while ours is based on simulated clinical case scenarios. Another important difference is that their application leads to some guidance advice including the possibility of self-care. Our application first determines the two potential guidance referrals: a referral to emergency departments or to primary care services. Then, it evaluates the two levels of urgency required for both categories: Emergency Level 1, Emergency Level 2, Primary Care Physician Level 1 and Primary Care Level 2. None of the advice given led to an abstention of a medical consultation ensuring better safety but probably also a higher demand for care, as previously reported [29].

### Factors affecting the performance of the tool

Few studies have focused on the factors that influence the performance of digital self-triage tools. In the present study, we investigated two main categories of factors suspected to influence the accuracy of the tool: the characteristics of the participants and the types of medical conditions screened through the clinical vignettes.

Regarding the clinical vignettes analysis, our results demonstrate lower agreement regarding neurological and dermatological diseases. Whether these findings can be generalized to the global patients’ self-triage process needs further investigation.

Second, based on our results, a lower degree of education is not significantly correlated with a lower accuracy when using the tool. Surprisingly, older age was not associated with lower performance of the tool. Those findings regarding education and age are encouraging for the further development of the application and its use by the global population equitably. The interest in digital-based healthcare information found on the internet does not seem to be correlated with a significant impact on the use of digital triage. While those data need to be confirmed in real-life settings, this statement unveils the possibility of extending the application to the global population regardless of age, degree of education and interest in digital-based healthcare. However, access to technological devices and the internet is still an unsolved problem in low-income populations and isolated or geriatric subgroups. In 2021 in Belgium, 8% of households still does not have access to the internet, a slightly better proportion than the 10% in 2019 [30, 31]. These figures are similar to the report from 2019 in which European countries demonstrated a 90% rate of families with effective access to the internet [32].

#### Overtriage and undertriage

The current tool provides some inappropriate advice regarding either the triage category, the level of care or the level of urgency. However, the decrease in accuracy is correlated with high percentages of overtriages, demonstrating that if the platform misdirects a patient, it is more frequently to a higher level of care or urgency. This finding provides a certain degree of reassurance regarding patient safety.

#### Comparison with Telephone Triage Services

In many countries, Telephone Triage Services are recognized as an efficient platform to manage unscheduled urgent care. One interesting element to determine is the comparison between the accuracy of self-triage platforms and Telephone Triage Services. In Belgium, two telephone triage services were implemented: the project SALOMON which took place from 2011 to 2019 in the Liège province and the nationwide 1733 number which was implemented more recently. The comparison between different services is a complex task because the setting and the methodology used to determine their respective accuracy are not necessarily identical. Two previous studies on the accuracy of these telephone services were conducted using a similar methodology than ours based on simulated scenarios. As previously mentioned, our self-triage platform sensitivity and specificity to distinguish the need for emergency care compared to primary care were estimated at 97% and 69% with an undertriage rate of 1.9% and an overtriage rate of 12.5%. The accuracy to determine the level of care and urgency was estimated at 68.4% with 9.2% of undertriages and 22.4% of overtriages. In the first preliminary study by *Morreel et al.,* researchers found that 1733 services show a sensitivity of 42% and a specificity of 92% with an overtriage rate of 39% and an undertriage rate of 26% when it comes to discriminate high urgent cases from low urgent cases [33]. The accuracy of their triage system to determine the correct urgency level among all the level’s categories was estimated at 35% [33]. In the study by *Brasseur et al.* regarding the SALOMON services, they demonstrated after a specific training a sensitivity of 99% and a specificity of 99% with an overtriage of 1% and an undertriage of 0.5% to guide the patient either to emergency care or primary care [16]. The accuracy of the SALOMON triage to determine the level of urgency was estimated at 98.5%. The interest for this comparison lies in the questioning about the accuracy of a given level of urgency obtained through a triage system which is used either by the patient itself, a non-clinical dispatcher, or a clinical dispatcher. Indeed, in the report by *Morreel et al.* the dispatcher is represented by a non-clinical trainee whereas *Brasseur et al.* founded their services through experienced triage nurses as the gold-standard dispatcher. Our results suggest that the patient itself can achieve a self-triage at least as accurate as the one furnishes by a non-clinical operator at a telephone triage helpline. One potential remark made by *Morreel et al.* regarding their study is the need for a more advanced training for their operators. This could represent a limitation for the present comparison. However, Nurse Telephone Triages Services seem to remain the most accurate orientation system compared to non-clinical dispatchers or the patient itself.

### Limitations and perspectives

This study provides promising results for developing this interactive platform. However, those need to be confirmed by further research based on real-life settings and larger cohorts to better represent all categories of potential users. Indeed, it is difficult to predict the proportion of patients whose self-perceived severity of illness will negatively influence the accuracy of the tool to provide an appropriate referral. Additionally, the stress generated by the unexpected disease could potentially play a role in the performance of the tool and the ability of the participant to use it appropriately. Health literacy was also not assessed and could potentially play a role in the ability to use such tools. Further research is required to evaluate the potential link between the accurate use of digital systems and the level of health literacy.

Whether all the different social subgroups will accurately use this kind of technological tool in the future is also an unpredictable statement [13] and the potential impact on the global healthcare system is currently poorly investigated. However, we hypothesize that this type of platform could play an interesting role in the patient’s education when confronted with an unplanned need for care.

Another essential point to discuss regarding self-triage tools is the structure of the application. The ODISSEE platform is based on decisional flowcharts with binary answers leading to specific advised destinations. Currently, several other different models of self-triage tools exist based on more sophisticated models driven by artificial intelligence (AI) [11, 34]. As AI-driven tools seem to present promising results in terms of safety, we should question the interest in developing classical algorithmic apps versus AI-driven apps.

Further research is needed to demonstrate the reproducibility and criterion validity of this type of digital solution. The major challenge for the future is to build safe interactive tools focusing essentially on quality but not profitability [29].

## Conclusion

Implementing innovative online patients self-assessment services could represent an original solution to manage the unscheduled care demand, especially if we consider the current propensity of patients to become involved in their health management. Two variables need to be considered while advising the patient: the level of care required and the level of urgency. The ODISSEE platform could represent a valuable strategy to guide patients in need of unscheduled care to the best level of care, as demonstrated by the obtained preliminary results. However, considering the level of urgency, the ODISSEE platform could offer a promising strategy subject to further improvements. Further investigations are needed to confirm these trends in real-life settings and evaluate the criterion validity of this tool. Furthermore, focusing both on the global performance of the tool, the patient’s characteristics, and the nature of the medical conditions that may affect triage accuracy is essential for the future as a question of safety and equity.

## Supplementary Information

**Additional file 1:Supplementary File S1.** Illustration of one clinical vignette used by the participants to assess the tool.

**Additional file 2: Supplementary File S2.** Distribution of the scenarios among the types of groups and the associated performance of the tool across the four triage categories, the levels of care and urgency.

## Data Availability

The main data are available in the manuscript and in Supplementary Files (Supplementary File S2). The full dataset is only available from the corresponding author upon reasonable request.
