# Supplementary material for: Patients’ self-triage for unscheduled urgent care: a preliminary study on the accuracy and factors affecting the performance of a Belgian self-triage platform
Source: BMC Health Serv Res. 2022 Sep 23;22:1199. doi: 10.1186/s12913-022-08571-5 (PMC9508742; doi:10.1186/s12913-022-08571-5)
Supplement: Supplementary file 1 — Additional file 1:Supplementary File S1. Illustration of one clinical vignette used by the participants to assess the tool. [file 12913_2022_8571_MOESM1_ESM.docx]

**Supplementary File S1. *Illustration of one clinical vignette used by the participants to assess the tool.***

| **Clinical Case Description** |
| --- |
| You connected to the application for the assessment of a head trauma.  You just hit a wood door at home, accidentally.  You didn’t collapse but you are in pain.  You have a wound on the scalp (see clinical picture).  You feel dizzy and nauseous.  Medical past :   - Gastroduodenal ulcus. - Myocardial Infarction.   Medications :   - Aspirin. - Omeprazole. |

| **Clinical Picture** |
| --- |
| **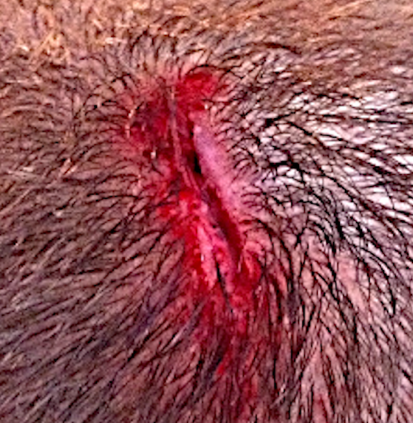** |
