# Supplementary material for: Patients’ self-triage for unscheduled urgent care: a preliminary study on the accuracy and factors affecting the performance of a Belgian self-triage platform
Source: BMC Health Serv Res. 2022 Sep 23;22:1199. doi: 10.1186/s12913-022-08571-5 (PMC9508742; doi:10.1186/s12913-022-08571-5)
Supplement: Supplementary file 2 — Additional file 2: Supplementary File S2. Distribution of the scenarios among the types of groups and the associated performance of the tool across the four triage categories, the levels of care and urgency. [file 12913_2022_8571_MOESM2_ESM.docx]

**Supplementary File S2.** *Distribution of the scenarios among the types of groups and the associated performance of the tool across the four triage categories, the levels of care and urgency.*

| Groups of scenarios | Triage category  N=750 | | | Level of care  N=750 | | | Level of urgency  n=642* | | |
| --- | --- | --- | --- | --- | --- | --- | --- | --- | --- |
|  | Appropriate | Under  triage | Over  triage | Appropriate | Under  triage | Over  triage | Appropriate | Under  triage | Over  triage |
| Dermatological diseases (n=84) | 52  (61.90%) | 16 (19,05%) | 16 (19,05%) | 75  (89.30%) | 2  (2.40%) | 7  (8.30%) | 52  (69.30%) | 14  (18.70%) | 9  (12.00%) |
| Trauma (n=185) | 127 (68.60%) | 22 (11.90%) | 36 (19.50%) | 163  (88.10%) | 5  (2.70%) | 17  (9.20%) | 127  (77.90%) | 17  (10.40%) | 19  (11.70%) |
| Digestive/Abdominal diseases and associated surgery complications (n=68) | 57  (83.80%) | 4  (5.90%) | 7 (10.30%) | 65  (95.60%) | 0  (0.00%) | 3  (4.40%) | 57  (87.70%) | 4  (6.15%) | 4  (6.15%) |
| Cardiac and pulmonary diseases (n=68) | 43  (63.20%) | 6  (8.80%) | 19 (28,00%) | 48  (70.60%) | 2  (2.90%) | 18  (26.50%) | 43  (89.60%) | 4  (8.30%) | 1  (2.10%) |
| Intoxication and psychiatric problems (n=51) | 38  (74.50%) | 6  (11.80%) | 7 (13.70%) | 47  (92.20%) | 0  (0.00%) | 4  (7.80%) | 38  (80.90%) | 6  (12.70%) | 3  (6.40%) |
| Neurological disorders (n=73) | 39  (53.40%) | 6  (8.20%) | 28 (38.40%) | 59  (80.80%) | 3  (4.10%) | 11  (15.10%) | 39  (66.10%) | 3  (5.10%) | 17  (28.80%) |
| Others (n=221)    *Minor diabetes complications*  *(n=7)*  *Pregnancy/delivery problems*  *(n=30)*  *Ocular disorders*  *(n=31)*  *Ear-nose-throat disorders*  *(n=23)*  *Frequent pediatric disorders*  *(n=23)*  *Non-traumatic articular conditions (n=54)*  *Problem involving >5 patients*  *(n=7)*  *Fever*  *(n=46)* | 157 (71.00%)  *6 (85.70%)*  *22 (73.40%)*  *21 (67.70%)*  *19 (82.06%)*  *16 (69.60%)*  *30 (55.60%)*  *7 (100%)*  *36 (78.30%)* | 9  (4.10%)  *0 (0%)*  *1 (3.30%)*  *0 (0%)*  *1 (4.30%)*  *1 (4.30%)*  *3 (5.50%)*  *0 (0%)*  *3 (6.50%)* | 55 (24.90%)  *1 (14.30%)*  *7 (23.30%)*  *10 (32.30%)*  *3 (13.10%)*  *6 (26.10%)*  *21 (38.90%)*  *0 (0%)*  *7 (15.20%)* | 185  (83.70%)  7 (100.00%)  23 (76.70%)  28 (90.30%)  20 (87.00%)  19 (82.60%)  39 (72.20%)  7 (100.00%)  42 (91.30%) | 2  (0.90%)  0 (0.00%)  0 (0.00%)  0 (0.00%)  0 (0.00%)  0 (0.00%)  1 (1.9%)  0 (0.00%)  1 (2.20%) | 34  (15.40%)  0 (0.00%)  7 (23.30%)  3 (9.70%)  3 (13.00%)  4 (17.40%)  14 (25.90%)  0 (0.00%)  3 (6.50%) | 157  (84.90%)  6 (85.70%)  22 (95.70%)  21 (75.00%)  19 (95.00%)  16 (84.20%)  30 (77.00%)  7 (100.00%)  36 (85.70%) | 7  (3.80%)  0 (0.00%)  1 (4.30%)  0 (0.00%)  1 (5.00%)  1 (5.30%)  2 (5.00%)  0 (0.00%)  2 (4.80%) | 21  (11.30%)  1 (14.30%)  0 (0.00%)  7 (25.00%)  0 (0.00%)  2 (10.50%)  7 (18.00%)  0 (0.00%)  4 (9.50%) |
| Overall | 513 (68.40%) | 69  (9.20%) | 168 (22.40%) | 642  (85.60%) | 14  (1.90%) | 94  (12.50%) | 513  (79.90%) | 55  (8.60%) | 74  (11.50%) |

Notes: (*) Sample size was adjusted after removing falsely classified cases by the participants.
